# Attitudes towards suicidal behavior in medical students of Lahore, Pakistan

**DOI:** 10.1192/j.eurpsy.2023.707

**Published:** 2023-07-19

**Authors:** S. Azeem, N. Imran, N. Afzal, Z. Jamil, I. I. Haider

**Affiliations:** ^1^Psychiatry, King Edward Medical University; ^2^Psychiatry, Mayo Hospital; ^3^Psychiatry, Fatima Memorial Hospital, Lahore, Pakistan

## Abstract

**Introduction:**

The increasing global suicide rates pose a considerable strain on healthcare professionals. Subsequently, their attitudes toward suicide prevention may influence suicide risk and management, affecting the quality of care.

**Objectives:**

To investigate the attitudes of Pakistani medical students toward suicide and its comparison with different sociodemographic factors.

**Methods:**

A total of 1392 undergraduate medical students belonging to all five years took part in the cross-sectional study conducted in September 2022. In addition to socio-demographic factors, participants were asked about their attitudes toward suicide on a 5-point Likert scale using the ATTS (Attitudes towards suicide) questionnaire. Questions explored competence, religion, experience, and views on suicidal behavior and its treatment. Data were analyzed by using SPSS 26.

**Results:**

The majority of respondents had no prior experience of looking after patients with suicide attempts (88.9%), the experience of having known someone who died by suicide (67.1%), or participation in suicide workshops (94.3%). Statistically significant items showed that males believed more strongly that suicide could be used to end suffering and would consider the possibility of doing it, revenge is the major driving factor, talking about suicide lessens its incidence, and people should have the right to take their own lives. Females more strongly believed that loneliness is the major driving factor, and that suicide is preventable. Preclinical students more strongly believed thought suicide was less justified, especially among young people, not a solution to end incurable illnesses, and that people should not have the right to take their own lives. 996 (71.6%) of respondents expressed their willingness to participate in workshops regarding suicide.

**Conclusions:**

Our study suggests that medical students have little experience in handling suicidal patients and vastly differ in their attitudes. There is a need for suicide management training and further study data to support these findings.

**Disclosure of Interest:**

None Declared

